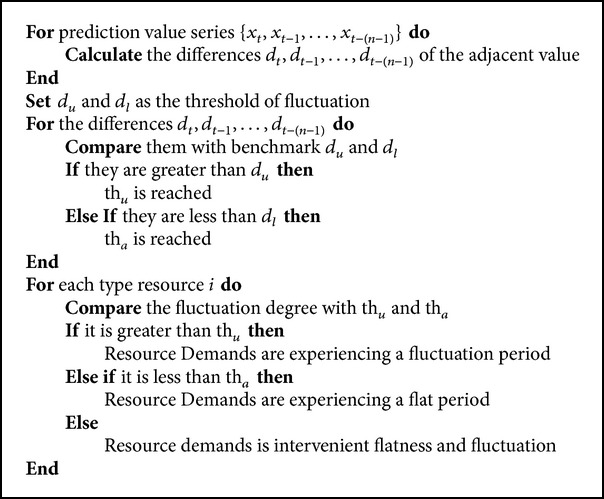# Self-Adaptive Prediction of Cloud Resource Demands Using Ensemble Model and Subtractive-Fuzzy Clustering Based Fuzzy Neural Network

**DOI:** 10.1155/2015/919805

**Published:** 2015-01-26

**Authors:** Zhijia Chen, Yuanchang Zhu, Yanqiang Di, Shaochong Feng

**Affiliations:** Department of Electronic and Optics, Mechanical Engineering College, Shijiazhuang 050003, China

## Abstract

In IaaS (infrastructure as a service) cloud environment, users are provisioned with virtual machines (VMs). To allocate resources for users dynamically and effectively, accurate resource demands predicting is essential. For this purpose, this paper proposes a self-adaptive prediction method using ensemble model and subtractive-fuzzy clustering based fuzzy neural network (ESFCFNN). We analyze the characters of user preferences and demands. Then the architecture of the prediction model is constructed. We adopt some base predictors to compose the ensemble model. Then the structure and learning algorithm of fuzzy neural network is researched. To obtain the number of fuzzy rules and the initial value of the premise and consequent parameters, this paper proposes the fuzzy *c*-means combined with subtractive clustering algorithm, that is, the subtractive-fuzzy clustering. Finally, we adopt different criteria to evaluate the proposed method. The experiment results show that the method is accurate and effective in predicting the resource demands.

## 1. Introduction

In cloud computing [1, 2], high accurate and efficient resource provisioning is an important aspect for maximizing the utility. In IaaS mode cloud computing [3], resources are allocated in the form of virtual machines which are composed of virtual hardware virtualized by hypervisor [4]. Users send requests to cloud center and try to obtain the resources in accordance with their demands. However, before cloud center provisioning these resources for users, some time may be needed to prepare and initialize the instance, i.e. the VMs. On the other hand, when the VM is running, resources dynamic adjusting is also needed to guarantee the QoS (quality of service). Some schemes do not consider the customer driven management, where resources have to be dynamically rearranged based on customers' demands [5]. The rearrangement of resources cannot take effect instantly and some time is needed, which leads to insufficiently providing the elastic management of resources [6]. Moreover, if the resources are not allocated properly, the performance of VMs may be restricted, or resources may be idle and wasted. This severely decreases the utility and meanwhile the QoS of cloud computing.

It stands to reason that resource provisioning in the cloud environment is influenced directly by performance predictions [7]. In order to know how to allocate resources beforehand, it is important to characterize users' demands and preferences accurately. To make an accurate prediction, this paper analyses the main factors that affect the prediction performance and proposes a prediction method that proves to be more accurate and effective.

The key contributions of this paper are listed as follows:

1. We make analysis of the characters of user demands and preferences. Corresponding models and solutions are researched.
2. Self-adaptive cloud resource demands prediction algorithm using fuzzy neural network is proposed. Besides historical data, the base predictors' output results are adopted by FNN with different weight.
3. Fuzzy* c*-means combined with subtractive clustering (i.e., subtractive-fuzzy clustering) algorithm is adopted to optimize the convergence features and learning speed.
4. The learning algorithm of fuzzy neural network is optimized with self-adjusting learning rate and momentum weight, which improves the robustness and the real-time performance.
5. To evaluate the prediction algorithm, some statistic indexes are introduced to compare with other algorithms, MSE (mean squared error), MAE (mean absolute error), and PRED(*x*).

## 2. Literature Review

### 2.1. Cloud Resource Demands Prediction

Researches on resource demand prediction are mainly focused on how to save energy [8], improve performance [7, 9], increase profit [5, 10, 11], and so on. To optimize resource management and task scheduling, Ramezani et al. [12] introduce a prediction method of VM workload patterns and VM migration time using fuzzy expert system. However, only a simple prediction model is depicted and the details are not explicated. By contrast, a fuzzy prediction method is given in [13] to model the uncertain workload and the vague availability of virtualized server nodes, using type-1 and type-2 fuzzy logic systems. Adopting fuzzy algorithm, the performance of prediction method is more robust but the accuracy is decreased. This method needs to be combined with another prediction method to realize high performance.

There are some methods predicting resource demands based on duration time. Reference [14] proposes an approach for long-term trend prediction using moving average method. To control jitter in a small range, it further improves the conventional moving averages method using standard deviations. This method mainly aims at long-term prediction and the short-term prediction is not mentioned.

To balance the performance and the system cost, some researches make efforts to maximize the system utility. In [15], fast up and slow down algorithm is introduced to maximize the performance while maintaining the stability.

As workload has an obvious nonlinear feature, many machine-learning algorithms have also been used to support its prediction. Neural network (NN) is introduced as a prediction method [16–19]. Combining with the typical predicting methods such as sliding window method [20], auto regression model [21], and exponential smoothing model [22], they have worked well in predicting the workload. To improve the inference ability, fuzzy neural network is introduced for predicting.

### 2.2. Fuzzy Neural Network

Fuzzy neural network is a combination of a fuzzy logic system and a neural network. It keeps the merit of each [23]. The algorithm is widely used in various applications such as pattern recognition, prediction, and system control [24–26]. In prediction area, it has been discovered to be more accurate than other conventional or soft computing techniques. In [27], neurofuzzy and neural network techniques are adopted to forecast the sea level in Darwin Harbor. It is proved that adaptive neurofuzzy inference system (ANFIS) is more effective in predicting than autoregressive moving average (ARMA) algorithm. References [28, 29] introduce FNN approach into energy consumption demands predicting. Results of [28] even show that the hybrid ANFIS model has better performance than ANN in terms of prediction accuracy. Some works [30, 31] use FNN algorithm in hydrological time series prediction. In the machine condition maintenance area, FNN algorithm is used to predict condition of the machine or the components [32, 33].

According to the literature reviewed above, FNN is used in many areas for predicting and the performance is fine. Unfortunately, in cloud resource demands predicting area, few researches have adopted FNN as predicting method. In this paper, we adopt FNN with self-adjusting learning rate and momentum weight as the core of the prediction system. To improve the performance, we use ensemble model and clustering algorithm to optimize FNN prediction system. Before the data is sent to the predictor, some preparation work needs to be done.

## 3. System Overview and Preparation for Prediction

### 3.1. System Overview

**Figure 1:**
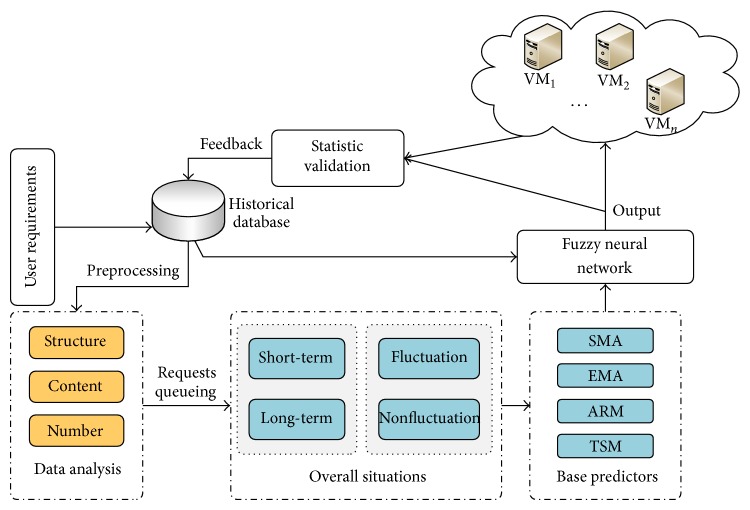
The overview of the prediction system.

Before prediction of user demands, we firstly analyze the user requests, including the utilization data structure, content, and number of historical resources. By analyzing the historical data, we may draw conclusions about user preference, demand description, and so forth. The short-term versus long-term and the fluctuation period versus flat period prediction are separately treated. Fluctuation-threshold (th_*u*_) and flat-threshold (th_*a*_) are defined to distinguish the flat period and fluctuation period. In different periods, different base prediction methods are adopted according to the characters, such as second moving average model (SMA), exponential moving method (EMA), autoregression model (ARM), and trend seasonality model (TSM). The output of the base predictors is sent to the fuzzy neural network as input. Fuzzy neural network uses the historical data and the base prediction value as training data, which improves the accuracy of the results. The output of fuzzy neural network is used to instruct the resource allocation in IaaS cloud center. Prediction results and the actual resource demands are evaluated using statistical analysis and different criteria. The evaluation results are fed back to the historical database to improve the prediction performance. The overview of resource demands prediction system is depicted in Figure 1.

### 3.2. Overall Situations of User Demands

As depicted in Figure 1, there are some opposite circumstances to be concerned, such as long-term demands and short-term demands and fluctuation period and nonfluctuation period. Fluctuation period is an abnormally violent vibration on a cloud resource over a period of time.

In user long-term resources requirements, there are some characters, as we summarize in the following: (1) the regularity is more obvious than in short-term. It is understandable that the long-term users may show some repetition regularity; (2) in the long running of the system, there may be some fluctuation periods and some flat periods. Coming to short-term requirements, the regularity may be not much obvious, and the fluctuation feature is more noticeable. Therefore, the long-term or short-term may be not conflicted with fluctuation or flat periods. For long-term data, we can on the one hand summarize the regularity. On the other hand, the flat and fluctuant periods should be distinguished. For short-term data, the regularity is not easy to figure out and the fluctuation should be processed. The prediction speed should be ensured as the short-term resources provision gives first place to quick response than other performance. The difference between short-term and long-term processing mainly lies in the fluctuation period processing. Hereby we discuss the fluctuation period and flat period, respectively.

#### 3.2.1. Flat Period Procession

**Figure 2:**
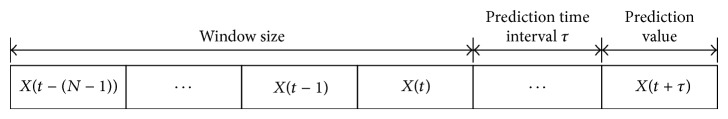
The model of sliding window.

Based on the characters of flat period, the second moving average (SMA) [34] is adopted, it can effectively reduce the lag deviation between prediction value and actual value. In this method, we define a sliding window whose input size is *N*, that is, *X* = [*x*_*t*_, *x*_*t*−1_,…, *x*_*t*−(*N*−1)_] over the historical time interval [*t* − (*N* − 1), *t*].  Figure 2 depicts the method model of sliding window with a window size *N*.

The predicted output value *x*_*t*+*τ*_ after a prediction time interval *τ* is the dependent variable set *X*, where *X* = [*x*_1_, *x*_2_,…, *x*_*N*_]. Then the relationship can be abstracted as follows:

(1) $$\begin{array}{c} x_{t+\tau }=f\left(X,\tau \right). \end{array}$$

The *i*th user's resource requirement prediction at time *t* + *τ* can be expressed as follows:

(2) $$\begin{array}{c} x_{t+\tau }\left(i\right)=a_{t}\left(i\right)+\tau b_{t}\left(i\right). \end{array}$$

In function (2), *x*_*t*+*τ*_(*i*) is the prediction value in time *t* + *τ*, *τ* is the time sequence number to be predicted. *a*_*t*_(*i*) and *b*_*t*_(*i*) satisfy the following constraint equation:

(3) $$\begin{array}{c} a_{t}\left(i\right)=2M_{t}^{\left(1\right)}\left(i\right)-M_{t}^{\left(2\right)}\left(i\right), \end{array}$$

(4) $$\begin{array}{c} b_{t}\left(i\right)=\frac{2}{N-1}\left[M_{t}^{\left(1\right)}\left(i\right)-M_{t}^{\left(2\right)}\left(i\right)\right]. \end{array}$$

In formula (3), *M*_*t*_^(1)^(*i*) and *M*_*t*_^(2)^(*i*) separately represent the first moving average value and the second moving average value at the *t*th time of the resource the *i*th user requested. *N* is the time period of moving average period. In addition, *M*_*t*_^(1)^(*i*) and *M*_*t*_^(2)^(*i*) can be expressed as follows:

(5) $$\begin{array}{c} M_{t}^{\left(1\right)}\left(i\right)=\frac{x_{t}\left(i\right)+x_{t-1}\left(i\right)+\cdots +x_{t-\left(N-1\right)}\left(i\right)}{N},\\ M_{t}^{\left(2\right)}\left(i\right)=\frac{M_{t}^{\left(1\right)}\left(i\right)+M_{t-1}^{\left(1\right)}\left(i\right)+\cdots +M_{t-1}^{\left(1\right)}\left(i\right)}{N}. \end{array}$$

Then the total number of the resources requested by all the *m* users is

(6) $$\begin{array}{c} x_{t+\tau }=\overset{m}{\underset{i=1}{\sum }}x_{t+\tau }\left(i\right). \end{array}$$

From the analysis above, we can see that the prediction value at the time *t* + *τ* is decided only by the values of the *N* periods' values at the time *t* and the total number of the users. And the prediction result can be calculated at time *t*.

#### 3.2.2. Fluctuation Period Procession

Exponential moving method (EMA) is an effective method for short-term prediction and particularly suitable for time series prediction of the nonseasonal effect owing to its quick responsiveness and weight decreases with time passed. Predicted values are calculated using smoothing constant *α*. The exponential moving average is expressed as follows:

(7) $$\begin{array}{c} x_{t+1}=\alpha X\left(t,N\right)+\left(1-\alpha \right)x_{t}. \end{array}$$

In (7), *X*(*t*, *N*) is the moving average value between the past time *t* − (*N* − 1) and the current time *t*. Then the time interval is *N*. *α* is the smoothing constant that can be calculated by *α* = 2/(*N* + 1). We can see that *α* confines to [0,1].

The EMA method gives a higher weight to the later measure value and lower weight to the earlier measure value. So the EMA method is able to response rather quickly to the fluctuations in a short-term demand and workload conditions [35]. However, there will be some delay introduced as the window size increases. Based on Andreolini and Casolari [36], in nonlinear load trackers, the polynomial orders should be properly selected. If the order is low (degree ≤ 2), then the function will not react quickly enough to load changes. If the order is high (degree ≥ 4), the function will be unnecessarily complex and some undesirable sparks will be introduced. The cost may be too expensive for a run-time context.

#### 3.2.3. The Identification of Flat Period and Fluctuation Period

Though we give different prediction methods according to the wiggle levels, it is difficult to know or identify the boundary of different wiggle levels in the overall situation. In this section, we define “fluctuation-threshold (th_*u*_)” and “flat-threshold (th_*a*_)” to distinguish the fluctuation and flat periods. Fluctuation-threshold is defined as the upper limit of the degree of vibration demand on cloud resource, while flat-threshold is the lower limit. In the last *n* period of time, if the difference *d*_*t*_ of prediction value *x*_*t*_ and *x*_*t*−1_ in series {*x*_*t*_, *x*_*t*−1_,…, *x*_*t*−(*n*−1)_} is greater than a certain value *d*_*u*_, then the th_*u*_ is reached. If the difference of *x*_*t*_ and *x*_*t*−1_ is less than a certain value *d*_*l*_, then the th_*a*_ is reached. For resource type *i*, the demands are experiencing a fluctuation period if the demand data in last *k* time satisfies the condition Deg_*i*_ ≥ th_*u*_, where Deg_*i*_ means the fluctuation degree of the prediction trend. th_*u*_ is the upper limit value. Type *i* resource demands are experiencing a flat period if the demand data in last *k* time satisfies Deg_*i*_ ≤ th_*a*_. If th_*a*_ ≤ Deg_*i*_ ≤ th_*u*_, the demands of resource *i* are intervenient flatness and fluctuation.

The above procedure can be illustrated by Pseudocode 1.

## 4. Resource Demands Prediction Using Optimized Fuzzy Neural Network

In order to predict the resource demands accurately and effectively, a prediction method with different individual base prediction models ensemble and fuzzy neural network is proposed in this section. With different base prediction models, different demands occasions can be estimated accordingly, and the most likely future outcome is able to be predicted. With the results of base predictors, the fuzzy neural network tends to present better predicting performance. To improve the learning ability, we use self-adjusting learning rate and momentum weight to optimize the learning procedure. Clustering algorithm is adopted to initialize the fuzzy inference rules of the FNN. The introduction of fuzzy neural network promises the robustness and accuracy of the prediction system.

**Figure 3:**
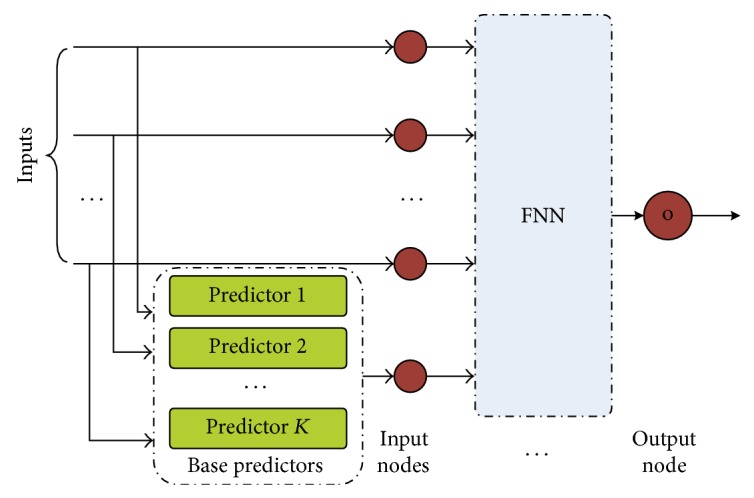
The structure of ensemble model and FNN based prediction model.

The core of the prediction model adopts a two-level structure, as shown in Figure 3. The first level is an ensemble model that contains different base predictor models. The output of the first level is sent to the second level, fuzzy neural network, which is responsible for optimizing the precision and the robustness of the prediction results.

### 4.1. Base Prediction Models

As we know, diversity is necessary for the survival and evolution of species ensemble model. So as for the performance of the prediction models, it is important to introduce the diversity to the prediction ensemble model. To guarantee the prediction performance; the base prediction models should be firstly selected. Besides the prediction models mentioned in Section 3, some other models are introduced. The guideline of choosing is based on the capacity and overheads.

#### 4.1.1. Autoregression Model

Autoregression model (ARM) is one of the linear models that are used for estimating the relationships between one dependent output variable *y* and one or more independent variables *x*_*i*_. It represents how the dependent value changes along with each independent variable changing. The fundamentals of the method are to treat the historical measurement data as a stochastic process which can be treated as a white noise driven filter. It is proved effective for predicting host load. The form of an AR model is

(8) $$\begin{array}{c} y=\alpha_{1}x_{t-1}+\alpha_{2}x_{t-2}+\cdots \alpha_{p}x_{t-p}+e_{t}, \end{array}$$

where *e*_*t*_ is white noise signal that contains all the unpredictable information in the past.

#### 4.1.2. Trend Seasonality Prediction Model

Trend seasonality model (TSM) represents a regularity that repeats periodically, which can be modeled by low order polynomials. To measure how the general cycle affects data value, we calculate a series of periodic indicators. Seasonality indicator demonstrates the offset between certain period average value and the overall average value. To get an accurate estimation of the indicator, each periodic value is calculated and compared with the total average value. The seasonal indicator Ind_*i*_ can be calculated by the equation Ind_*i*_ = avg_*i*_/avg, where avg_*i*_ is the average value of the *i*th period and avg is the total value. The periodic data are generated by the cloud users' resource requirements. Based on the indicators, the future resource requirements are predicted by two steps: (1) compute the future trend level by using a polynomial equation with order two; (2) introduce the seasonal influence by multiplying the trend level by indicators.

#### 4.1.3. Moving Average Method

By judging whether the monitoring data crosses over the moving average, moving average method (MAM) predicts the future trend. MA(*N*) is a moving average of monitoring data series *R*_*i*_ with length of *N*, expressed as equation MA(*N*) = ∑_*i*−(*N*−1)_^*i*^(*R*_*i*_/*N*), s.t. *N* ≤ *i*. If the monitoring data cross over the moving average upward, it indicates that an ascending trend is coming. While if the monitoring data cross over the moving average upward, it indicates that a descending trend is coming.

### 4.2. Fuzzy Neural Network

In fuzzy neural network, we use neural network to evolve the fuzzy inference rules. We consider a multi-input single-output (MISO) fuzzy model which consists of *l* rules. The *i*th if-then rules of fuzzy inference system can be expressed as follows.

*Rule i*. If *x*_1_ is *A*_1_^(*i*)^ and *x*_2_ is *A*_2_^(*i*)^ and … *x*_*n*_ is *A*_*n*_^(*i*)^, then *y* is *B* ^(*i*)^.

In the rules, *A*_*i*_, *B* ^(*i*)^, and *M*_*i*_ are the linguistic labels (e.g., high or low) associated with the node functions. The “if part” (antecedent) is fuzzy in nature, while the “then part” (consequent) is a crisp function of an antecedent variable.

#### 4.2.1. The Structure of FNN

**Figure 4:**
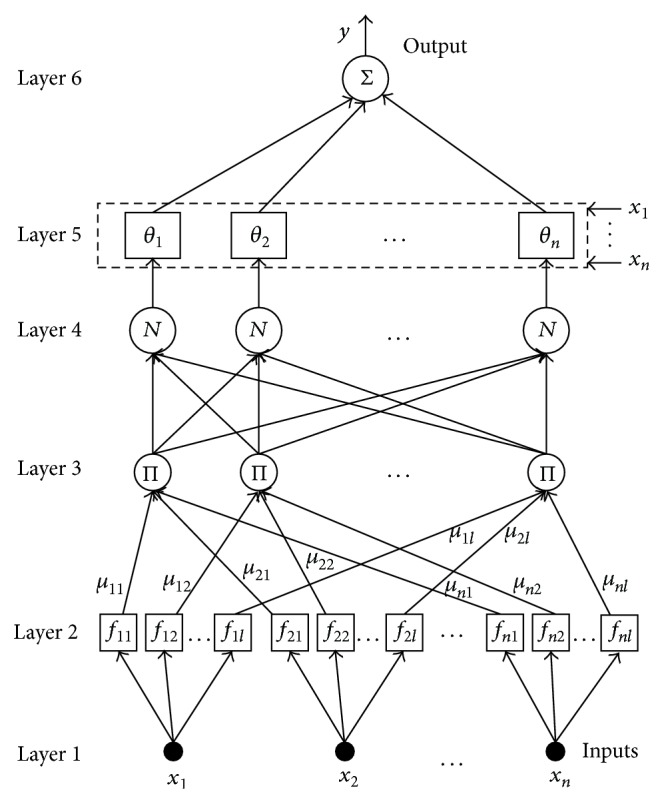
The architecture of FNN.

To give a full description to the FNN without loss of generality, we construct the FNN as shown in Figure 4. net_*j*_^(*k*)^ means the net input of *j*th node in the *k*th layer. *y*_*j*_^(*k*)^ is the output of the *j*th node in the *k*th layer. *x*_*j*_^(*k*)^ is the input of the *j*th node in the *k*th layer. Here the relation between *x* and *y* is *y*_*j*_^(*k*)^ = *x*_*j*_^(*k*+1)^. The meanings and function relations of each layer are discussed in the following.

Layer 1 is known as the input layer. Nodes in this layer transmit the input data to the next layer directly. Input data is abstracted as *X* = {*x*_1_, *x*_2_,…, *x*_*n*_}. The relationship between input and output can be expressed as follows:

(9) $$\begin{array}{c} {\text{net}}_{j}^{\left(1\right)}=x_{i}^{\left(1\right)},\;i=j,\;y_{j}^{\left(1\right)}={\text{net}}_{j}^{\left(1\right)}. \end{array}$$

Layer 2 is known as the fuzzification layer. In this layer, every node performs the calculation of a Gaussian membership function and specifies the degree to which the given input *x*_*i*_ satisfies the quantifier *A*_*i*_. Consider

(10) $$\begin{array}{c} \text{ne}{\text{t}}_{j}^{\left(2\right)}=-\frac{1}{2}{\left(\frac{x_{i}^{\left(1\right)}-m_{ij}}{\sigma_{ij}}\right)}^{2},\\ y_{j}^{\left(2\right)}=\mu_{A_{j}}\left(x_{j}\right)=\exp\left(\text{ne}{\text{t}}_{j}^{\left(2\right)}\right). \end{array}$$

Here, *m*_*ij*_ and *σ*_*ij*_ are the center and the width of the Gaussian function of the *j*th term of the *i*th input variable, respectively. Both *m*_*ij*_ and *σ*_*ij*_ are adjustable parameters.

Layer 3 is the fuzzy inference layer. The fuzzified results of the individual scalar functions of each input data are aggregated. All potential rules of the input data are formulated by applying fuzzy intersection, which means product of data. Thus, a product operation denoted as ∏ is performed to obtain the output of this layer. Consider

(11) $$\begin{array}{c} \text{ne}{\text{t}}_{j}^{\left(3\right)}=\underset{i}{\prod }x_{ij}^{\left(3\right)}=\underset{j}{\prod }y_{j}^{\left(2\right)}\;\;y_{j}^{\left(3\right)}=\text{ne}{\text{t}}_{j}^{\left(3\right)}. \end{array}$$

The output of layer 3 represents the firing strength of the rules.

Layer 4 is the normalization layer. Each node in layer 4 is labeled with *N* which denotes “normalization.” The ratio of the *i*th rule firing strength to the total firing strengths is calculated in this layer. The relationship between input data and output data is expressed as follows:

(12) $$\begin{array}{c} \text{ne}{\text{t}}_{j}^{\left(4\right)}=\frac{x_{i}^{\left(4\right)}}{\sum_{i}x_{i}^{\left(4\right)}}\;\;y_{j}^{\left(4\right)}=\text{ne}{\text{t}}_{j}^{\left(4\right)}. \end{array}$$

The output of this layer is known as normalized firing strength.

Layer 5 and layer 6 are known as the defuzzification layers. In layer 5, every node in this layer is labeled with *θ*_*i*_. The node function is as follows:

(13) $$\begin{array}{c} \text{ne}{\text{t}}_{j}^{\left(5\right)}=\theta_{i}x_{i}^{\left(4\right)}\;\;y_{j}^{\left(5\right)}=\text{ne}{\text{t}}_{j}^{\left(5\right)}. \end{array}$$

Here *θ*_*i*_ is the adjustable weight parameter. Parameters in this layer are known as consequent parameters.

In layer 6, there is only one node labeled with ∑. It is used to compute the output of the fuzzy neural network. The output is the summation of all the incoming data from layer 5. Consider

(14) $$\begin{array}{c} \text{ne}{\text{t}}_{1}^{\left(6\right)}=\underset{i}{\sum }x_{i}^{\left(6\right)}\;\;y_{1}^{\left(6\right)}=\text{ne}{\text{t}}_{1}^{\left(6\right)}. \end{array}$$

#### 4.2.2. The Learning Procedure of FNN

After the structure of FNN is constructed, we use error back propagation method to adjust the parameters *θ*_*l*_, *m*_*ij*_, and *σ*_*ij*_. The objective function is defined as

(15) $$\begin{array}{c} E=\frac{1}{2}e^{2}=\frac{1}{2}{\left(\hat{y}-y_{1}^{\left(6\right)}\right)}^{2}, \end{array}$$

where $\hat{y}$ is the instructor signal. The error propagates from the output layer back to the input layer. To clearly analyze the back propagation procedure, we combine layer 5 and layer 6 as one.

In the defuzzification layer (layer 5), the evolution of error is expressed as follows:

(16) $$\begin{array}{c} \delta_{1}^{\left(5\right)}=-\frac{\partial E}{\partial \text{ne}{\text{t}}_{1}^{\left(5\right)}}=\hat{y}-y_{1}^{\left(5\right)}=e, \end{array}$$

(17) Δθi=−η∂E∂θi=−η∂E∂net1(5)·∂net1(5)∂θi=η·δ1(5)·xi(5)=η·δ15·yi4=η·δ15·∏ixi.

In layer 4, the differential error of node *j* is

(18) $$\begin{array}{c} \delta_{j}^{\left(4\right)}=-\frac{\partial E}{\partial \text{ne}{\text{t}}_{j}^{\left(4\right)}}=-\frac{\partial E}{\partial \text{ne}{\text{t}}_{j}^{\left(5\right)}}\cdot \frac{\partial \text{ne}{\text{t}}_{j}^{\left(5\right)}}{\partial \text{ne}{\text{t}}_{j}^{\left(4\right)}}=\delta_{1}^{\left(4\right)}\cdot \theta_{j}. \end{array}$$

In layer 3, *δ*_*j*_^(3)^ is

(19) δj(3)=−∂E∂netj3=−∂E∂netj(5)·∂netj(5)∂netj(4)·∂netj(4)∂netj(3)=δj4·∑jnetj3−netj3∑jnetj32.

In layer 2, *δ*_*j*_^(2)^ is

(20) δj(2)=−∂E∂netj(2)=−∂E∂netj5·∂netj5∂netj4·∂netj4∂netj3·∂netj3∂netj2=δj3·∏j=1j≠iμij,Δmij=−η∂E∂mij=−η∂E∂netj(2)·∂netj(2)∂mij  =2ηδj2·exp⁡−xi−mij2σij2·xi−mijσij2,Δσij=−η∂E∂σij=−η∂E∂netj(2)·∂netj(2)∂σij=2ηδj2·exp⁡−xi−mij2σij2·xi−mij2σij3.

The update parameters are

(21) $$\begin{array}{c} \theta_{l}\left(t+1\right)=\theta_{l}\left(t\right)+\eta_{1}\cdot \Delta \theta_{l}\left(t\right),\\ m_{ij}\left(t+1\right)=m_{ij}\left(t\right)+\eta_{2}\cdot \Delta m_{ij}\left(t\right),\\ \sigma_{ij}\left(t+1\right)=\sigma_{ij}\left(t\right)+\eta_{3}\cdot \Delta \sigma_{ij}\left(t\right). \end{array}$$

#### 4.2.3. Self-Adjusting Learning Method and Momentum Weight

In (17), the learning rate *η* determines the convergence speed of the neuron network. If *η* is small, the changes of synapse weight in the iterative computation procedure will be small and the locus of the weight space becomes smooth. However, the learning rate is decreased. If *η* is too large, the learning rate will increase but the network may become unstable and may cause wobble of the weights. To optimize the convergence speed and stability of the neural network, a momentum term can be included in (17), and it is expressed as follows:

(22) $$\begin{array}{c} \Delta \theta_{k}^{\left(l\right)}=\eta \delta_{k}^{\left(l\right)}{\overset{-}{y}}_{k}^{\left(l\right)}+\lambda \Delta \theta_{k}^{\left(l-1\right)}, \end{array}$$

where *λ* is the momentum constant. Equation (22) introduces the preceding Δ*θ*_*k*_^(*l*−1)^ into the procedure of calculating Δ*θ*_*k*_^(*l*)^. The use of the momentum constant is a minor revise for refreshing the weight. However, it makes some advantages for the learning speed of the algorithm.

In addition, we adopt a “progressive-increase” and “conservative-decrease” method to adjust the learning rate *η*. If the error declines in the training procedure, we consider the modification direction to be right and a larger adjusting variable *k* is used. If the error is becoming bigger, we regard that the modification is excessive and the adjusting step needs to be slowed down and a smaller value is assigned to variable *k*. Meanwhile, the former modification should also be abandoned. The method is shown in the following function:

(23) $$\begin{array}{c} \eta \left(i+1\right)=\left\{\begin{array}{cc} k_{\text{inc}}\eta \left(i\right) & E\left(i+1\right)<E\left(i\right)\\ k_{\text{dec}}\eta \left(i\right) & E\left(i+1\right)>E\left(i\right). \end{array}\right. \end{array}$$

The variable *i* means the learning steps. *k*_inc_ and *k*_dec_ are, respectively, the increase factor and the decrease factor.

### 4.3. Optimized Clustering Method

#### 4.3.1. Fuzzy c-Means Clustering

In FNN, the construction of fuzzy if-then rules is difficult. The improper rule set may result in bad prediction results. Recently, a number of different approaches have been used for designing fuzzy if-then rules based on clustering, gradient algorithms [37], genetic algorithms [38], fuzzy* c*-means clustering [39], and subtractive clustering [40]. Fuzzy clustering is an efficient technique for constructing the antecedent structures. The aim of clustering methods is to identify a certain group of data from a large data set, such that a concise representation of the behavior of the system is produced. Each cluster center can be translated into a fuzzy rule for identifying the class. In this paper, the fuzzy* c*-means clustering technique is used for structuring the premise part of the fuzzy system.

By analyzing the membership degree of sample data, the fuzzy* c*-means algorithm clusters partitions data set of different classes. Consider that there are *n* objects *X* = {*x*_1_, *x*_2_,…, *x*_*n*_}. Fuzzy* c*-means partitions them into *c* fuzzy clusters, where *c* confines to 1 < *c* < *n*. The centroids of the clusters are *Z* = {*z*_1_, *z*_2_,…, *z*_*c*_}. The form of fuzzy clustering of objects is a fuzzy rule set matrix *μ* with *n* rows and *c* columns, where *n* and *c* are the total number of data objects and the number of clusters separately. *μ*_*ij*_ indicates the degree of association or membership function of the *i*th object with the *j*th cluster. The characters of *μ* are shown in the following:

(24) μij∈0,1 0<∑i=1nμij<n ∑j=1cμij=1i=1,2,…,n; j=1,2,…,c.

The optimization objective function of FCM algorithm is

(25) $$\begin{array}{c} J\left(\mu_{ij},z_{i}\right)=\overset{n}{\underset{i=1}{\sum }}\overset{c}{\underset{j=1}{\sum }}\mu_{ij}^{m}d_{ij}^{2}\;\;d_{ij}=\left\|x_{i}-z_{j}\right\|. \end{array}$$

In the above equation, *m* is the exponent weight and it controls the fuzziness of the clusters and *d*_*ij*_ is the Euclidian distance between objective *x*_*i*_ and the centroid *z*_*j*_. By minimizing *J*(*μ*_*ij*_, *z*_*i*_), the centroid of the *j*th cluster can be calculated using the following equation:

(26) $$\begin{array}{c} z_{j}=\frac{\sum_{i=1}^{n}\mu_{ij}^{m}x_{i}}{\sum_{i=1}^{n}\mu_{ij}^{m}}. \end{array}$$

The membership degree matrix can be calculated by the following equation:

(27) $$\begin{array}{c} \mu_{ij}=\frac{1}{\sum_{k=1}^{c}{\left(d_{ij}/d_{ik}\right)}^{2/\left(m-1\right)}}. \end{array}$$

#### 4.3.2. Subtractive Clustering

From the discussion above, we can see that FCM is sensitive to isolated data. As the sum of the membership degree has to be 1, the results may be not good if the sample data is not ideal. Besides, the cluster centroids of FCM are initialized stochastically. If the initial value is not properly selected, the convergence may be affected and local convergence may happen. Thus, FCM relies on initial centroids greatly. Moreover, the diversity of membership function may lower the convergence speed.

To improve the FCM algorithm, we introduce the subtractive clustering as a complement. Subtractive clustering is unsupervised clustering, in which the number of clusters for input data points is determined by the clustering algorithm. The subtractive clustering does not need to define the number of the clusters. The results may be used for initializing the centroids of FCM algorithm. It assumes that each data is a potential cluster centroid. Based on the data density index of the potential centroid data, we select the data that has the highest density as the centroid. The procedure is concluded as follows.

(1) Calculating the density index of each data *x*_*i*_
  (28) $$\begin{array}{c} D_{i}=\overset{n}{\underset{k}{\sum }}\exp\left[-\frac{{\left\|x_{i}-x_{k}\right\|}^{2}}{{\left(r_{a}/2\right)}^{2}}\right]. \end{array}$$

The clustering radius *r*_*a*_ is determined by the following equation:

(29) $$\begin{array}{c} r_{a}=\frac{1}{2}\underset{k}{\min}\left\{\underset{i}{\max}\left\|x_{i}-x_{k}\right\|\right\}. \end{array}$$

Data beyond the radius affects little to the density index. We firstly choose the data *x*_*c*1_ that has the highest density index *D*_*c*1_ as the first cluster centroid. Then the data in the radius *r*_*a*_ is removed from the potential centroid data set.

(2) We use the following equation to modify the density index of each data:
  (30) $$\begin{array}{c} D_{i}=D_{i}-D_{c1}\exp\left[-\frac{{\left\|x_{i}-x_{c1}\right\|}^{2}}{{\left(r_{b}/2\right)}^{2}}\right]. \end{array}$$

s.t. *r*_*b*_ > *r*_*a*_. A neighborhood with lower density index is defined by the above equation. The aim is to keep one centroid away from others so that the clusters may be distinct from others. As the density index of the data that is close to the first cluster centroid *x*_*c*1_ is much lower, the potential to be the centroid is also decreased.

(3) By calculating the density index of the remaining data, the next centroid is obtained. If the constraint equation (31) is achieved, we regard *D*_*ck*_ as the centroid of cluster *C*_*k*_:
  (31) $$\begin{array}{c} \frac{D_{ck}}{D_{c1}}<\delta . \end{array}$$

Here, 0 < *δ* < 1 is the constraint parameter that decides the number of the cluster centroid. Through (31), we can see that the number of clusters is inversely proportional to *δ*. Furthermore, the identification sequence of centroids is decided by density index. The higher the density index is, the earlier the centroid emerges, and the proper centroid probability becomes greater.

#### 4.3.3. The Combination of Fuzzy c-Means and Subtractive Clustering

This section introduces a clustering algorithm that combines FCM with subtractive clustering method. We obtain the cluster centroids and number through subtractive cluster. This can effectively improve the convergence speed of FCM and the probability of local convergence is decreased.

The procedure can be described as follows.

**Step 1 .**

Set the parameters, including neighborhood radius *r*_*a*_ and *r*_*b*_, fuzzy exponent weight *m*, and comparison parameter *δ*.

**Step 2 .**

Calculate the number and centroids of clusters through subtractive method.

**Step 3 .**

Use (25) and (27) to calculate the objective function and the membership degree.

**Step 4 .**

Verify if the termination constraints are achieved. If ‖*J* ^(*K*+1)^ − *J* ^(*k*)^‖ < *ε* or the maximum iteration is achieved, the operation process terminates. Otherwise, turn to Step 5.

**Step 5 .**

Update *k* with *k* + 1 and turn to Step 3. Use (25) and (27) to calculate new cluster centroids and membership degree.

### 4.4. Validation Criterions

To evaluate the performance of the prediction system, we use a series of metrics [20] including MAE (mean absolute error), MSE (mean squared error), and PRED(*x*).

#### 4.4.1. MAE and MSE

MAE is the criterion of measuring the mean deviation between the prediction output and the actual output. MAE can be calculated by the following function:

(32) $$\begin{array}{c} \text{MAE}=\frac{1}{n}\overset{n}{\underset{i=1}{\sum }}\left|{\hat{y}}_{i}-y_{i}\right|, \end{array}$$

where ${\hat{y}}_{i}$ is the actual output and *y*_*i*_ is the prediction value. *n* is the number of the data series. The smaller the value of MAE is, the more accuracy the prediction method is.

MSE represents the energy of the mean error. MSE can be expressed as in the following:

(33) $$\begin{array}{c} \text{MSE}=\frac{1}{n}\overset{n}{\underset{i=1}{\sum }}{\left({\hat{y}}_{i}-y_{i}\right)}^{2}. \end{array}$$

#### 4.4.2. PRED(*x*)

PRED(*x*) is the proportion of the prediction data number whose relative error falls within ±*x* × 100% to the whole data number. Take PRED(5) as an example, according to formula [14], we define the relative error as

(34) $$\begin{array}{c} {\tilde{e}}_{i}=\frac{y_{i}-{\hat{y}}_{i}}{{\hat{y}}_{i}},\;i=1,2,\ldots ,n. \end{array}$$

*i* represents the series number of the output data series. The number of all the relative errors that meet the condition $-5\%\leq {\tilde{e}}_{i}\leq 5\%$ is supposed as *k*_(5)_. The whole number is *n*. Then PRED(5) is defined as

(35) $$\begin{array}{c} \text{PRED}\left(5\right)=\frac{k_{\left(5\right)}}{n}. \end{array}$$

The index PRED(5) represents the fitness of the prediction model. If the value is close to 1.0, it indicates a good fit of the prediction model.

### 4.5. Feedback Control

To optimize the performance of the resource demands prediction system, we introduce the feedback control [41] into the system. In each prediction cycle, the feedback controller sends the actual resource demands and prediction results to historical database. Specially, the demands value is specified in fine-grained form, including the elements in data structure vector *P*_*i*_. In addition, the validation indexes of MAE, MSE, PRED(10), and so forth are also processed in the controller. The feedback controller sends corresponding value to the historical database.

## 5. Experimental Evaluation

**Figure 5:**
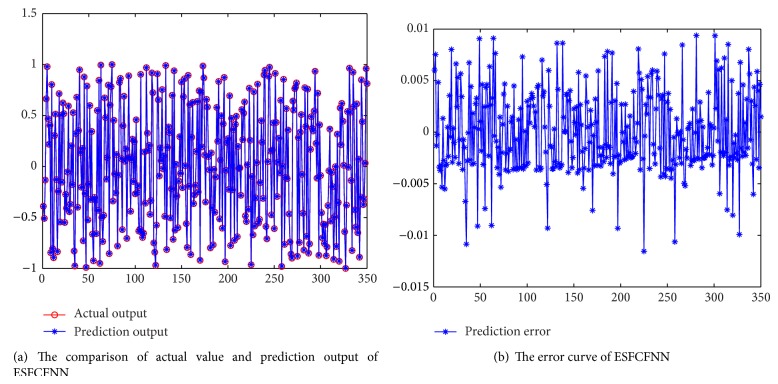
The training results and error of ESFCFNN.

In this section, experiments are conducted to validate the proposed prediction method. When we predict the fine-grained resource demands, the method of each kind of resource is similar to others. Here we do not distinguish resource type, and we use network traffic as the representation. From [42], we sample 400 days network visit traffic data. We use anterior 350 days traffic data as training data and posterior 50 days traffic data as test data. The training effect is shown in Figure 5. In Figure 5(a), the blue curve represents the prediction value and the red curve represents the actual value. The two curves fit with each other. In Figure 5(b), we can see that the overall effect is promising. The maximum error is controlled within −1.1%~+1%. The training results are accurate.

**Figure 6:**
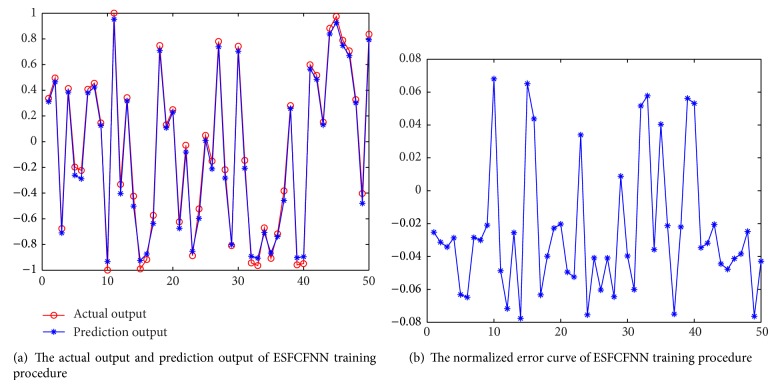
The test results and error of ESFCFNN.

The test data is used to verify the prediction method. The test results are shown in Figure 6. In Figure 6(a), the blue curve is the prediction value and the red curve is the actual value. We can see that the most of the two curves almost overlap with each other.  Figure 6(b) shows the prediction error. We can see that the maximum normalized error is 8%. Most of the normalized error data falls within −8%~+8%. The prediction results are acceptable.

**Figure 7:**
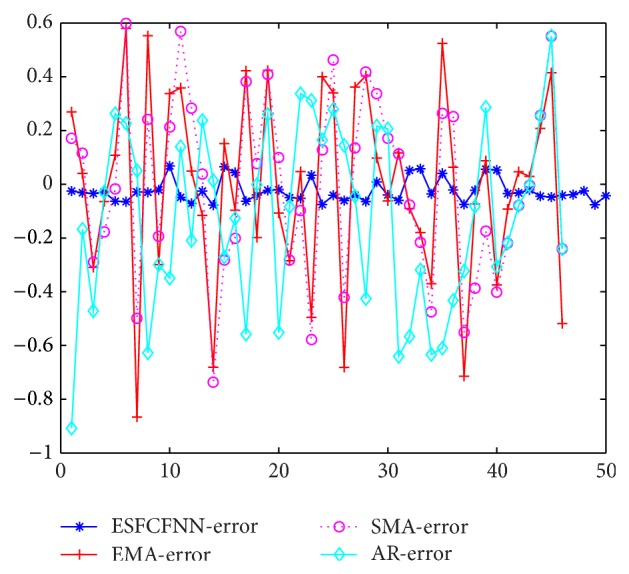
Normalized prediction error using different methods.

The prediction error using different methods is compared with each other and the results are depicted in Figure 7. In Figure 7, the performances of EMA, SMA, AR, and ESFCFNN methods are evaluated. The difference between different predicting methods is shown intuitively. We can see that the prediction error using ESFCFNN prediction method is apparently decreased. The results of ESFCFNN method are more accurate than other methods. The performance of ESFCFNN is optimized greatly.

**Figure 8:**
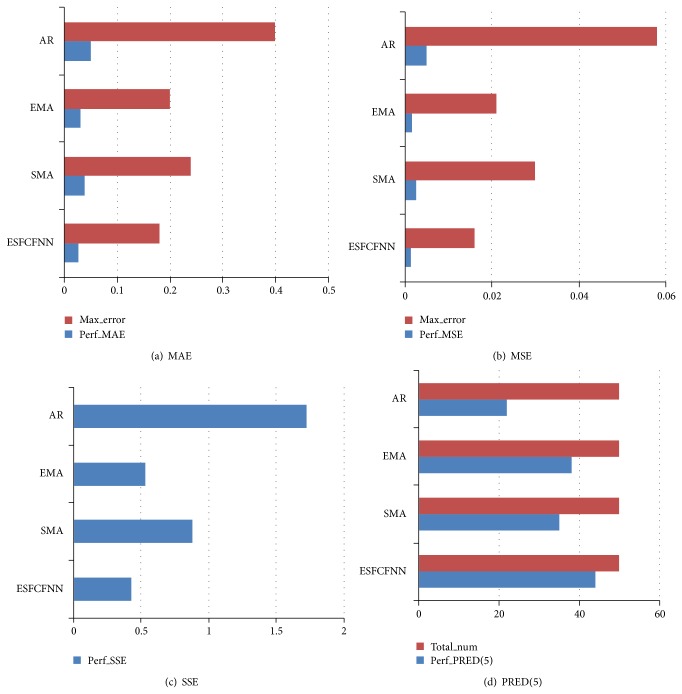
Prediction error indexes comparison of different methods.

According to the main criteria we defined from formulas (32) to (35), we test some base prediction methods including SMA, EMA, AR, and ESFCFNN. The results are shown in Figure 8. In Figure 8(a) we can see that both the mean average error in regularity and the max error of the ESFCFNN method are small. The performances of EMA and SMA are close to ESFCFNN. So as for the MSE and SSE, the ESFCFNN takes advantages of the other predictors' merits and realizes self-adaption and robustness. Finally, Figure 8(d) shows the number of errors that falls in 5%. The red columniation of Figure 8(d) is the total number, which are 50. The blue columniation is the number that falls in 5%. From the above analysis, we can conclude that the prediction results of ESFCFNN method are more accurate and this method is promising for predicting users' resource demands.

**Figure 9:**
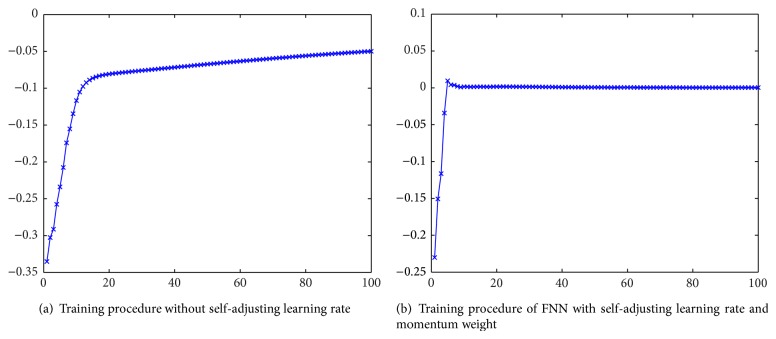
The comparison of the training performance of different learning algorithms.

Figure 9(a) depicts the training procedure without self-adjusting learning rate. After 100 training cycles, the error *P*_*a*_ of FNN is approximately 0.048. The error is very large.  Figure 9(b) depicts the performance of FNN with self-adjusting learning rate and momentum weight. After 100 training cycles, the error *P*_*b*_ reaches 0.0015. The ratio of *P*_*a*_ to *P*_*b*_ is *r* = *P*_*a*_/*P*_*b*_ = 0.048/0.0015 = 32.

The performance is much better after using self-adjusting learning rate and momentum weight. Moreover, the convergence speed after using self-adjusting learning rate is improved. The training error falls down to 0.05 within 10 steps when using self-adjusting learning rate. However, if the self-adjusting learning rate algorithm is not adopted, the convergence speed is slowed down. More than 85 steps are needed before error falls down to 0.05. From above analysis, we can see that the performance of FNN with self-adjusting learning rate and momentum weight is improved greatly.

**Figure 10:**
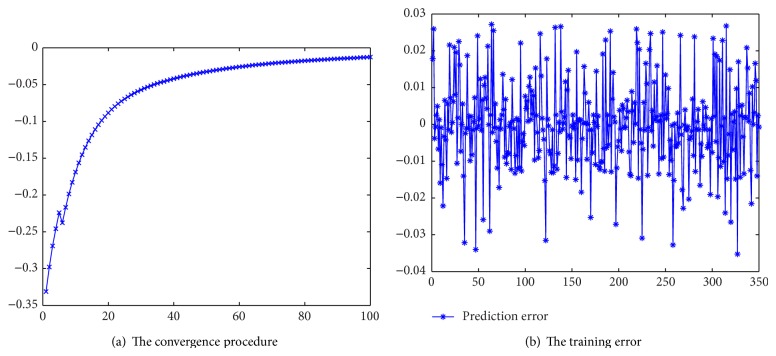
The performance without clustering.

In Figure 10, the performance of FNN without using clustering algorithm is depicted.  Figure 10(a) shows the convergence procedure. We can see that the convergence speed is slowed down. After more than 30 steps, the error falls down to 0.05. While Figure 9(b) shows that, with clustering algorithm, this procedure only needs less than 10 steps.  Figure 10(b) shows the training error of FNN without using clustering algorithm. Compared with Figure 5, the training error of FNN without using clustering algorithm is greater. From the above comparison, the performance is improved using clustering algorithm.

## 6. Conclusions

To improve the performance of resource provision and resource utilization, this paper proposed a cloud resource demands self-adaptive predicting method using ensemble model and subtractive-fuzzy clustering based fuzzy neural network, which is called ESFCFNN for short. We discuss the structure of the prediction system. Users' preferences are analyzed to reduce the amount of calculation. Then the base prediction model is introduced into the system. The results are sent to FNN with self-adjusting learning rate and momentum weight as the inputs. To optimize the convergence performance of FNN, fuzzy-subtractive clustering algorithm is proposed. Fuzzy-subtractive algorithm is composed with fuzzy* c*-means clustering algorithm and subtractive clustering algorithm. We evaluate the prediction system using statistic criteria including MAE, MSE, SSE, and PRED(5). The results show that ESFCFNN can effectively improve the prediction performance.

Though the method this paper proposes is promising in improving the performance, the system is complex. As we can see, there are two prediction layers. The time delay may be increased. In future, the improvement of efficiency is the main point of the research. We would also test the method in the real cloud computing system in future.

## Figures and Tables

**Pseudocode 1:**